# Clinical epidemiology and outcomes of ventilator-associated pneumonia in critically ill adult patients: protocol for a large-scale systematic review and planned meta-analysis

**DOI:** 10.1186/s13643-019-1080-y

**Published:** 2019-07-20

**Authors:** John Mark M. Gutiérrez, Annabelle R. Borromeo, Allan L. Dueño, Emmanuel D. Paragas, Rosanta O. Ellasus, Russel S. Abalos-Fabia, Jerry A. Abriam, Arnel E. Sonido, Monina A. Hernandez, Alain Jason A. Generale, Roberto C. Sombillo, Mary Grace C. Lacanaria, Mae M. Centeno, Jose Reinhard C. Laoingco, John Anthony A. Domantay

**Affiliations:** 1 School of Advanced Studies, Saint Louis University, Baguio, Philippines; 2 Department of Nursing, College of Applied Medical Sciences, Shaqra University, Shaqra, Saudi Arabia; 3 Division of Epidemiology, Department of Clinical Laboratory Science, College of Applied Medical Sciences, Shaqra University, Shaqra, Saudi Arabia; 4 Zone 3, Philippine Nurses Association, Quezon City and Marikina City, Philippines; 5 University of the Philippines Open University, Los Baños, Philippines; 6 Department of Nursing, West Liberty University, West Liberty, USA; 7 Institute of Community Health and Allied Medical Sciences, Don Mariano Marcos Memorial State University, Agoo, Philippines; 8 School of Nursing, Massey University, Palmerston North, New Zealand; 9 Clinical Research Support Office, Children’s Hospital Los Angeles, Los Angeles, USA; 10 Delos Santos Medical Center, Quezon City, Philippines; 11 School of Nursing, Saint Louis University, Baguio, Philippines; 12 Baylor Scott & White Health, Dallas, USA; 13 Graduate School of Nursing and Allied Medical Sciences, Holy Angel University, Angeles, Philippines; 14 School of Medicine, Saint Louis University, Baguio, Philippines

**Keywords:** Clinical epidemiology, Critical care nursing, Meta-analysis, Predictive analytics, Prognostic review, Risk assessment, Risk factors, Systematic review, Ventilator-associated pneumonia

## Abstract

**Background:**

An increasing number of studies have investigated the clinical epidemiology and outcomes of ventilator-associated pneumonia (VAP) in intensive care units. However, these findings have not been clearly defined in broad subgroups of mechanically ventilated adults. Hence, this protocol for a systematic review and meta-analysis is designed to better understand the clinical and epidemiological features of VAP in these patient populations by establishing its overall prognosis of and risk factors for morbidity and mortality and to determine the differences in clinical and economic outcomes between VAP and non-VAP patients.

**Methods:**

This present review will systematically search available full-text articles without date and language restrictions and indexed in PubMed, CENTRAL, CINAHL, Web of Science, and EMBASE databases. In addition, reference lists and citations of retrieved articles and relevant medical and nursing journals will be manually reviewed. Supplementary search in other databases involving trials, reviews, and grey literatures, including conference proceedings, theses, and dissertations, will be performed. Study investigators will be contacted to clarify missing or unpublished data. All prognostic studies meeting the pre-defined eligibility criteria will be included. The study selection, risk of bias assessment, data extraction, and grading of the quality of evidence will be carried out in duplicate, involving independent evaluation by two investigators with consensus or a third-party adjudication. The degree of inter-rater agreement will be calculated using the kappa statistic. For meta-analysis, dichotomous and continuous outcome measures will be pooled using odds ratios and standardized mean differences with 95% confidence intervals, respectively. The Mantel-Haenszel or inverse variance methods with random effects model will be used as a guide for analysis. The heterogeneity of each outcome measure will be assessed using both *X*^2^ and *I*^2^ statistics. In addition, sensitivity and subgroup analyses will be performed to ensure consistency of pooled results. The review protocol described herein is in accordance with the PRISMA-P standards.

**Discussion:**

The investigation of the epidemiological profiles, prognostic factors, and outcomes associated with VAP is critical for the identification of high-risk groups of mechanically ventilated patients and evaluation of possible clinical endpoints. This may provide substantial links for improved VAP prevention practices targeting modifiable risk factors. Implications for future research directions are discussed.

**Systematic review registration:**

PROSPERO CRD42017048158

**Electronic supplementary material:**

The online version of this article (10.1186/s13643-019-1080-y) contains supplementary material, which is available to authorized users.

## Background

Critically ill patients admitted to intensive care units (ICUs) require endotracheal intubation and mechanical ventilation (MV) to maintain airway patency, prevent aspiration, and improve oxygenation. Such supportive interventions are integral components of critical care and are considered as the gold standards for airway and ventilation management. However, both endotracheal intubation [1] and MV [2] are associated with numerous complications and may pose substantial risks among ICU patients. Evidence shows that prolonged duration of endotracheal intubation and MV may result to one or more of the following: susceptibility to laryngeal dysfunction, airway mucosal trauma, venous thromboembolism, lung injury caused by either over inflation or atelectasis, raised intracranial tension, and cardiovascular-related complications [1–4]. In addition, protracted use of these supportive measures may further predispose ICU patients to acquiring healthcare-associated infections, such as nosocomial pneumonia [2, 4]. This condition is the second most frequently occurring nosocomial infection in the ICU, affecting 14 to 27% of all hospitalized patients [5–7]. Of these, 24 to 86% are associated with MV [4, 5, 8]. This type of nosocomial infection is referred to as *ventilator-associated pneumonia* (VAP), a subtype of pneumonia that occurs 48 h following intubation and initiation of mechanical ventilatory support in patients who had no pre-existing lung infection upon admission [9].

Several VAP etiologies related to patient susceptibility have been discussed in existing literature [10], yet the definite mechanism of infection remains unclear because of a wide range of factors associated with VAP development. However, two pathophysiological processes have been proposed. These include colonization of pathologic microorganisms in the respiratory and digestive tracts and microaspiration of subglottic secretions into the dependent airways [11]. To further address this issue, an accumulating number of observational and interventional studies have linked several etiologies and prognostic factors to VAP development. These epidemiological findings have been summarized and integrated for the prevention and management of VAP in care for patients requiring MV [12, 13]. However, an a priori planned effort, performed by the principal investigator or PI (JMG), which involves electronic search of previously registered and published reviews and meta-analyses suggest that the risk factors associated with VAP development and the predictors of patient outcomes of morbidity (including prolonged duration of MV and increased ICU/hospital length of stay [LOS]) and mortality have not been thoroughly investigated in broad subgroups of mechanically ventilated adult ICU populations. Moreover, studies that investigate the epidemiology and clinical outcomes of VAP vary among patients requiring MV and across geographical locations [14]. Although VAP is considered to be a multifactorial condition, in recently conducted meta-analyses, different study investigators have identified few significant risk factors for VAP in critical care settings. The results vary among these studies and were only isolated to certain ICU patients, such as in neonatal [15], pediatric [16], and cardiac surgical populations [17, 18]. In addition to the mentioned preliminary search, the PI has found a previous meta-analysis that has attempted to shed light on the issue of risk factors associated with VAP in a notable number of mechanically ventilated adults; however, the said evidence focused mainly on VAP recurrence [19].

As per findings, there are no large-scale review studies that have been done thus far to address questions of clinical uncertainty between risk factors and the initial episode of VAP development, including the predictors of morbidity and mortality among the heterogeneous critically ill adult populations. Understanding these epidemiological features including the clinical and economic outcomes between subgroups of ICU populations is critical to help clinicians and policymakers in making appropriate decisions toward evidence-based preventive efforts. Therefore, explicitly identifying high-risk subgroups of mechanically ventilated patients is imperative for safe and effective implementation of infection control measures against VAP.

### Aims

The primary aim of this present review is to investigate the clinical epidemiology of VAP by establishing the overall prognosis of and risk factors for morbidity and mortality outcomes associated with this condition. For the secondary aims, the review investigators will cluster patients into groups, according to the presence (VAP group) or absence of VAP (non-VAP group), to compare the durations of MV, ICU/hospital LOS, mortality outcomes, microbiological findings, and antibiotic and hospitalization costs. Such investigations are critical to target specific prognostic factors for modification and further understand the impact of VAP on clinical and economic outcomes in broadly defined subgroups of critically ill adult populations.

## Methods

### Design

This protocol for a large-scale systematic review and meta-analysis was based on a recommended methodology [20] and followed the Preferred Reporting Items for Systematic review and Meta-Analysis for Protocols (PRISMA-P) 2015 guidelines (see Additional file 1) [21, 22] and was registered with the International Prospective Register of Systematic Reviews (PROSPERO) on 09 May 2017 (registration number: CRD42017048158). The latest registered protocol revisions were made on 09 October 2018.

### Eligibility criteria

Table 1 summarizes the review eligibility criteria. Below are the detailed descriptions of the inclusion and exclusion criteria:

**Table 1:** Eligibility criteria

Types of study designs	Any of the following VAP prognostic studies (published or unpublished) with corresponding control groups (non-VAP patients/survivors):
	Interventional study
	RCT or clinical trial
	Observational study
	Prospective cohort study or prospective analysis
	Retrospective cohort study or retrospective analysis
	Nested case-control study
	Case-control study with similar patient groups
	Case-control study with unclear or non-equivalent controls.
Types of participants	Any of the following adult (15 years or older) ICU population requiring MV:
	Cases: patients with VAP/non-survivor group/patients with clinical failure
	Controls: patients without VAP/survivor groups/patients with successful treatment
Types of exposures	Any of the following exposures:
	Host- or patient-related factors
	Treatment- or intervention-related factors
	Device-related factors
	Personnel-related factors
	Environmental-related factors
	Others (time-dependent factors, any ICU adverse events)
Types of outcome measures	Primary outcome measure
	Initial episode of microbiologically confirmed VAP (as defined by study authors)
	Any of the following secondary outcome measures:
	VAP as a predictor of ICU mortality (as reported by study authors)
	ICU mortality associated with VAP (as reported by study authors)
	ICU mortality associated with VAP in RCT studies
	ICU mortality associated with VAP in matched studies
	ICU mortality associated with clinically and microbiologically confirmed VAP
	In-hospital mortality associated with VAP (as stated by study authors)
	Duration of MV (time, measured in days, from initiation to discontinuation)
	ICU LOS (time, measured in days, from admission to ICU discharge or death)
	Hospital LOS (time, measured in days, from admission to hospital discharge or death)
	Microbiological findings by VAP-associated microorganisms (microbiological profile)
	Cost of antibiotic treatment of VAP (currency, in US dollar)
	Hospitalization costs (currency, in US dollar)
Types of ICU settings	No restrictions imposed
Types of languages	No restrictions imposed
Timing	Any of the following study endpoints: time to VAP, removal of critical ill patients from MV or planned extubation.
	No time restrictions for follow-up and retrospective analysis. However, patients must be intubated/ventilated for at least 48 hours.

*ICU* Intensive care unit, *LOS* Length of stay, *MV* Mechanical ventilation, *RCT* Randomized controlled trial, *US* United Stated, *VAP* Ventilator-associated pneumonia

#### Types of studies

The present review will include all possible prognostic studies (published or unpublished) with corresponding comparison group reporting a clear VAP definition and any of the following epidemiological indexes: VAP occurrence, etiology, and risk factors for and clinical outcomes of VAP-related morbidity and mortality. Clinical outcomes, by the definition, involve patient mortality, duration of MV, ICU/hospital LOS, microbiology, and antibiotic or health care costs. The review will consider prognostic studies utilizing any of the following study designs: randomized controlled trial (RCT), prospective cohort analysis, retrospective cohort analysis, nested case-control study, case-control study with similar patient groups, and case-control study with unclear/non-equivalent controls. The review anticipates a large number of prognostic factors derived from a body of observational evidence. A clinical trial or RCT is regarded because personnel- or intervention-related prognostic factors associated with patient morbidity (i.e., VAP, prolonged MV, increased hospital or ICU stay) are more likely to be reported in experimental design. Studies not meeting the aforementioned designs will be excluded, as well as studies with unclear methodology and unavailable data for risk calculation.

#### Types of participants

Study participants include heterogeneous adult (15 years or older) ICU patients requiring MV. The minimum age was based on the definition of the World Development Indicators regarding adult mortality rate [23]. Further, participants will be classified into two groups according to the following outcomes: VAP group and non-VAP group. For studies investigating VAP mortality, participants will be classified as survivor (i.e., patients without evidence of clinical failure) and non-survivor groups (i.e., patients who subsequently died during the course of ICU/hospital admission). In the present review, the following populations will be excluded: ICU patients not receiving endotracheal intubation or MV, non-ICU patients requiring respiratory support, pediatric populations including neonates, patients with recurrent VAP during the course of hospitalization, hypothetical cohorts, and non-VAP groups with different or insufficient definition.

#### Types of exposures

A variety of clinical exposures, which pertain either to contact with a disease-causing factor or the amount of factor that impinges upon a subgroup of patients, are essential components of risk assessment and predictive analytics for VAP prevention. This present review hypothesizes that specific host- or patient-related, intervention- or treatment-related, device-related, personnel-related, environmental-related, and other-related risk factors play a critical role in the development of VAP and have a significant impact on outcomes of morbidity and mortality in mechanically ventilated adult ICU patients. In order to identify exposure-related factors that increase the risk of VAP in critically ill adult patients, this present review will investigate the presence (or absence) of VAP-related morbidity and mortality and compare these clinical outcomes between two groups of mechanically ventilated patients: exposed and non-exposed. It will classify exposures as described in studies according to broad categories: host- or patient-related, treatment- or intervention-related, device-related, personnel-related, environmental-related factors, and others. Host- or patient-related risk factors are defined as the characteristics of patients (e.g., age, gender, admission diagnosis, comorbid conditions, immunosuppression) that are associated with VAP development. Intervention- or treatment-related risk factors are defined as care-related exposures (e.g., intubation, MV, blood transfusion, re-intubation, mobilization protocol) that may increase the probability of acquiring nosocomial pneumonia in mechanically ventilated patients. Device-related factors are potential sources of exposures (e.g., types of endotracheal tubes [ETTs], mechanical ventilator, pre-hospital airway devices, suction tubes, oral airways, ventilator circuits) that may also contribute to VAP development. Personnel-related risk factors refer to the staffing (e.g., nurse-patient ratio) or the compliance rates of clinicians to infection control measures (e.g., use of gloves, hand hygiene adherence), which are considered vital in VAP prevention and control. Environmental-related risk factors (e.g., types of hospitals, types of unit pathogens, fall-winter season) are defined as the characteristics in patients’ environmental conditions that increase their likelihood of acquiring VAP in ICU. Other related risk factors may refer to adverse events (i.e., unplanned extubation or self-extubation by patient, aspiration episodes, and complications following ICU admission, such as upper GI bleeding, sepsis, oxygen desaturation episodes, organ failure, and death) or time-dependent factors (i.e., durations of injury prior to admission, intubation, and MV; ICU/hospital LOS).

#### Types of outcome measures

Critically ill patients will be followed up for the development of initial episode of microbiologically confirmed VAP until extubation for more than 48 h, ICU discharge, or death. This event of interest will be regarded as the primary outcome measure. Secondary outcomes include duration of MV, ICU/hospital LOS, microbiological findings, antibiotic and hospital costs, and VAP mortality (ICU and/or in-hospital). Studies with clinically defined VAP outcome measures will be excluded. Clinically defined VAP studies may be regarded if study investigators only used a conventional diagnostic method in any of the following: clinical criteria (e.g., abnormal changes in body temperature [> 38 °C or < 35.5 °C], ratio of arterial blood oxygen tension to the concentration of inspired oxygen [PaO_2_/FiO_2_], presence or absence of purulent tracheobronchial secretions, and abnormal white blood cell count [> 10000/mm^3^ or < 4000/mm^3^]), radiologic findings (e.g., showing recent and persistent infiltrate on chest radiograph), and clinical pulmonary infection scores (CPIS). The present review will not consider clinical criteria with radiologic findings as primary bases for VAP diagnosis due to its inherent clinical limitations and issues on sensitivity and specificity [24]. The limitations of CPIS were also taken into account, as patients with composite CPIS score ≥ 6 may or may not have positive tracheal aspirate culture. However, this review will consider prognostic evidence combining multiple methods of VAP diagnosis, including CPIS and the new definition of ventilator-associated event (VAE) [25], provided that study investigators had explicitly reported microbiological confirmation (i.e., isolation of at least one pathogenic microorganism in significant bacterial counts obtained via bronchoscopic and non-bronchoscopic investigations) for all case patients.

#### Types of settings

All critically ill adults admitted in the different critical care settings will be included for this review. Restrictions by type of ICU setting or country of origin will not be imposed. Non-ICU settings (e.g., wards, long-term health care facilities) will be excluded.

#### Types of language

This present review will consider peer-reviewed articles written in languages other than English where translation is possible with the resources of our respective institution.

#### Timing

There is no maximum length of follow-up or retrospective analysis that will be imposed for this review. However, to fulfill the criteria for VAP, case patients must be mechanically ventilated for ≥ 48 h at the time of enrollment; hence, studies not meeting this MV cutoff value will be excluded. This time window is crucial in distinguishing primary from secondary infections or in categorizing pneumonia as community-acquired or ventilator-associated. The durations of endotracheal intubation/MV prior to VAP development (time to VAP) and ICU/hospital LOS prior to death (time to death) will be determined.

### Search methods and strategies

To identify potential search terms and retrieve the best set of results possible, a pre-determined MEDLINE search strategy was constructed using the PICO (patients, interventions, comparators, outcomes) equivalents: patients, exposures of interests, comparison, outcomes, and study design (see Additional file 2). All relevant search terms used with this approach were checked by a university librarian. Adjustment of search terms such as alternative keywords or phrases relative to specific prognostic factors for VAP and study designs will be adapted as necessary to capture all the results that might be relevant to the search topic. Detailed descriptions of search methods and strategies are outlined below:

#### Electronic searches

The present review will exhaust all available full-text articles (without date and language restrictions) indexed in the following major scientific databases:

- MEDLINE (via PubMed)
- The Cochrane Central Register of Controlled Trials (CENTRAL) (via The Cochrane Library)
- Cumulative Index to Nursing and Allied Health Literature (CINAHL) (via EBSCO*host*)
- Web of Science (via Saudi Digital Library)
- Excerpta Medica dataBASE (EMBASE)

The pre-determined MEDLINE search strategy will be adopted in searching relevant articles in all other databases. Modifications to indexing terms (e.g., Medical Subject Headings, field tags) for other databases will be applied as necessary.

#### Reference lists

All reference lists of the included original articles and some relevant reviews will be manually screened to reduce the possibility of excluding important citations.

#### Citation tracking

The present review will perform citation tracking using the Scopus**®** database to capture relevant citations that were possibly missed during the initial search. The PI (JMG) has chosen Scopus**®** because it is considered as the largest abstract and citation database of peer-reviewed literature, including scientific medical and nursing journals, books, and conference proceedings.

#### Handsearching

To further find additional articles, a select number of medical and nursing journals focusing on infection control and intensive-critical care will be electronically handsearched from date of inception to present issue (see Additional file 2). PubMed database will be utilized for this purpose using the following search strategy: “<journal National Library of Medicine (NLM) title abbreviation>”[jour] AND ventilator-associated pneumonia. Such strategy is crucial to highlight evidence containing “ventilator-associated pneumonia” or its related terms (e.g., VAP, ventilator-acquired pneumonia, ventilation-associated bacterial pneumonia, intubation-related pneumonia, nosocomial pneumonia, device-associated healthcare-acquired pneumonia) in the title, abstract, and/or keywords. Further, if the volume/issue is not indexed in the PubMed database, the journal’s website with relevant articles matching the abovementioned search terms will be manually explored.

#### Alternative searches

An additional search in supplemented databases using trial (ClinicalTrials.gov) and review (PROSPERO) registers will be carried out to identify ongoing or recently completed trials and systematic reviews, respectively. In addition, relevant studies in grey literatures, including conference proceedings and studies indexed in OpenGrey/ProQuest dissertations and theses databases, will be checked. Study investigators or corresponding authors will be contacted to clarify missing or unpublished data.

### Data collection

#### Selection of studies

The strategic study selection process will be performed in duplicate to identify relevant citations, screen titles, or abstract, retrieve and evaluate full-text articles against the pre-determined eligibility criteria, include eligible studies for review, and generate study codes accordingly. This process will be initially performed by the PI (JMG) and subsequently by six trained RAs (AD, EPJ, RE, RAF, JA, and AS). Each RA will be assigned to specific databases including other resources (e.g., reference lists, Scopus**®**, journals) to independently cross-validate the abovementioned procedures following a search strategy decision algorithm (see Additional file 3). Any disagreements will be resolved by discussion until a consensus is reached. The PRISMA flow diagram will be used to illustrate the evidence search and study selection process (see Additional file 3) [21].

#### Assessment of risk of bias in included studies

To evaluate the methodological quality of eligible studies, the QUIPS (Quality In Prognosis Studies) tool will be utilized (see Additional file 4) [26]. This tool was designed to support the evaluation of prognostic factor studies, wherein the validity and risk of bias are assessed based on the following six key domains: (1) study participation (selection bias), (2) study attrition (attrition bias), (3) prognostic factor measurement (misclassification or measurement bias), (4) outcome measurement (bias due to inadequacy of outcome measurement), (5) study confounding (bias due to confounding factors), and (6) statistical analysis and reporting (bias due to inadequacy of the statistical analysis). The decision for each of these six potential bias domains will be rated as high, moderate, or low risk of bias, considering all the responses to the prompting items to inform risk of bias judgment. To generate the overall risk of bias, the review investigators will take into account the bias ratings across the six important key domains. For instance, if a study is rated as having “low risk of bias” for all or the most important of the six key domains, then a summary assessment of risk of bias will be regarded as a study with “low risk of bias.” This selected definition will also be applied in studies rated as having “high” or “moderate risk of bias” for all or the most important of the six key domains.

The aforementioned risk of bias assessment will also be performed in duplicate, involving independent evaluation by two reviewers (1 investigator [PI] and 1 external reviewer) with consensus for each included study. A third person (AB) will be consulted if disagreements cannot be resolved. A total of three independent external reviewers (MH, AJG, and RS) will be part of this process. Each will be given assigned studies by the PI (JMG) to reach the target review timeline. To illustrate the decisions for each risk of bias domain in every included study, a risk of bias summary 3 × 3 table will be created and this will be used for kappa calculations.

#### Data extraction and management

For each included study, data from both published and unpublished reports will be independently extracted in duplicate by the PI (JMG) and one data extractor (RAF or RE) using a structured, pilot-tested, author-made data extraction form (see Additional file 4) with the following variables:

- General characteristics of the study;
- Sociodemographic and clinical characteristics of subjects;
- Outcome definition (VAP diagnostic criteria);
- Onset and incidence or prevalence of VAP among exposed and non-exposed subjects;
- Numbers of VAP-associated microorganisms;
- Risk factors associated with VAP and clinical outcomes of morbidity and mortality;
- VAP clinical outcomes including patient mortality, duration of MV, and ICU/hospital LOS; and
- Antibiotic treatment and hospitalization costs.

For data management, the PI (JMG) developed a platform using Microsoft**®** Office Excel, Mac 2011 version to provide a transparent document summary of the study selection process. First author name, study title, journal, database, publisher, relevance, number of articles needing full-text evaluation, reasons for exclusion, and number of included articles will be documented. All meta-analytic data with dichotomous outcomes will be entered in a 2 × 2 table following the 7-step validation process: The PI (JMG) will enter four important data items (total numbers of exposed patients [step 1], cases [step 2], enrolled patients [step 3], and exposed cases [step 4]), check all the totals matching the published study (step 5), check the calculated effect size (step 6), and assess the validity of entered data (step 7) to ensure completeness and accuracy. For data with continuous outcome measures including pooled data set, similar procedures will be applied as appropriate. These will be validated by one RA (RAF or RE). Any disputes will be resolved by discussion.

#### Dealing with missing data

In handling missing or unpublished data, the study investigators will be consulted if applicable. A request to obtain missing or incomplete data from study investigators will be forwarded. All requests will be documented and included in the *List of studies awaiting assignment*.

### Data analysis

All patient clinical information, including the outcomes of VAP-related morbidity and mortality, will be pooled using frequencies, percentages or proportions, and means (± SDs). Descriptive epidemiology will be employed for incidence density, prevalence rate, and proportional crude mortality. To estimate the number of patients with VAP, all new and pre-existing cases in a given period of time will be included and divided to the total population during the same time period. To estimate the proportional mortality attributable to VAP, relative risk increase (RRI) will be calculated using the following formula: crude mortality_cases_ − crude mortality_controls_/crude mortality_controls_ [27]. Chi-square test will be used to assess the difference between variables in VAP and non-VAP patients as appropriate. Kappa statistic will be performed to quantify the degree of inter-rater agreement between two reviewers. The strength of agreement will be interpreted in one of six categories using the accepted approach [28]: poor, slight, fair, moderate, substantial, or almost perfect. The SPSS Statistics Software (IBM Corp, Armonk, NY) for Windows, version 21.0, will be used for all above analyses.

To combine all relevant studies investigating VAP epidemiological outcomes, a meta-analysis will be performed using Review Manager software (RevMan, Copenhagen: The Nordic Cochrane Centre, The Cochrane Collaboration) for Mac OS X, version 5.3. In this analysis, study weights for dichotomous and continuous outcomes will be generated using Mantel-Haenszel (M-H) and inverse variance (IV) methods, respectively. Due to the inclusion of case-control studies with dichotomous outcomes, odds ratio (OR) with 95% confidence interval (CI) will be calculated to identify risk factors imposed on VAP development and clinical outcomes of morbidity and mortality. Odds ratio will also be used to investigate VAP attributable mortality among patients with VAP. In addition, standardized mean difference (*d*) with 95% CI will be calculated for continuous outcomes to compare the duration of MV, ICU/hospital LOS, and antibiotic and hospitalization costs between VAP and non-VAP patients. Pooled estimates with 95% CI for each outcome measure will be calculated using random effects model. Forest plots will be constructed to illustrate a summary of findings from individual prognostic studies. Such analyses are essential to accurately estimate the overall association between the abovementioned variables. A statistically significant association will be considered if *p* value is < .05. A summary of the research questions and sample planned data analyses is presented in Additional file 2: Table S7. However, if prognostic data will not be suitable for meta-analysis (i.e., presence of important methodological heterogeneity, missing data) following a detailed evaluation of the eligible studies, a summary of findings using tabular presentation will be generated to qualitatively synthesis prognostic evidence.

#### Assessment of heterogeneity

Since VAP outcomes differ between studies, this present review anticipates the presence of significant heterogeneity underlying broad epidemiological evidence. The methodological and clinical heterogeneity may be attributed to different study designs, sampling errors, sample size, characteristics of study populations, and the like. The heterogeneity between included studies will be assessed using both the *X*^2^ test for homogeneity and *I*^2^ statistic. Significant statistical heterogeneity will be regarded if the *X*^2^ test for homogeneity obtains a *p* value threshold of < .10. Methods to investigate explanations of heterogeneity of results between studies are discussed below.

#### Subgroup analysis

If adequate data pertaining to patient age, type of illness severity, type of country (by development status), type of study population, type of onset, type of causative microorganisms, methods of diagnosis, and presences (as opposed to absences) and types of immune suppression, prior or initial antimicrobial therapy, VAP protocol are reported from the included studies, the review investigators plan to carry out a priori subgroup analyses between studies to explain inconsistency between important subgroups. The following are definitions of subgroup analyses with corresponding categories, considering the study hypotheses and pre-specified direction of effect:

- Age. Age is one of the significant factors for VAP, as patients with advanced age (≥ 75 years old) are more likely to acquire healthcare-associated infections (including VAP) following hospital admission compared to other age groups (e.g., adults, young adults). An a priori subgroup analysis by age (in years) is planned for the following categories: ≤ 30, 30 to 60, 60 to 75, and ≥ 75.
- Type of illness severity. The severity of illness is defined as the extent of patient’s physiologic decompensation or organ system derangement measured by ICU severity scoring systems at admission, as patients with higher severity of illness index have higher risk of developing poor clinical outcomes (e.g., VAP, mortality). An a priori subgroup analysis by type of illness severity is planned for the following categories (based on previously defined cutoff points): according to acute physiology and chronic health evaluation (APACHE) II scores, < 20, 20 to 29, and ≥ 30 [29] and according to simplified acute physiology scores (SAPS) II, ≤ 33, < 33 to ≤ 45, < 46 to ≤ 58, and > 58 [30].
- Type of country by development status. This may refer to a country’s economic or trade status, as resources play a critical role on the incidence and risk factors for VAP development. The concept of resource inadequacy is one of the reasons why VAP rates can be substantially different in other settings. For instance, in developing countries, a larger number of VAP incidence are reported with varying risk factors involved than developed countries. An a priori subgroup analysis by country classification is planned for the following categories (based on previously defined status): developing, in transition, and developed [31].
- Type of patient population. This may refer to medical, surgical, trauma, and mixed ICU patients, as trauma patients might have greater need of mechanical ventilation compared to other ICU populations, therefore increasing their degree of exposure to VAP potential factors. An a priori subgroup analysis by type of patient population is planned for the following categories: medical (including coronary), surgical, cardiac surgical (including cardiovascular surgical), neurosurgical, trauma surgical, trauma (non-surgical), burn, neurological, oncological, and mixed ICU patients (including all patient categories).
- Type of disease onset. Onset (in days) may refer to the initial manifestations of VAP as examined by the physician intensivist or other outcome assessors. The pathogenesis and risk factors associated with VAP are dependent on the disease onsets. For this review, a definite cutoff period will be provided to categorize VAP onset based on the mean cumulative reports of primary studies, as VAP onset definitions may differ between studies. An a priori subgroup analysis by type of onset is planned for the following categories: early-onset and late-onset VAP.
- Type of causative microorganisms. This may refer to the pathologic agent causing VAP, as the attributable risk of VAP appears to vary with infecting pathogens, considering microbial virulence. This review will not consider *Candida* as a pathogen in our immunocompromised population. An a priori subgroup analysis by type of causative microorganisms is planned for the following categories (based on their potential to develop multidrug resistance): high-risk and low-risk microorganisms.
- Method of diagnosis. This refers to the different methods of VAP diagnosis, considering clinical criteria and radiological findings with microbiological confirmation (i.e., positive culture with a significant concentration of microorganisms) obtained via bronchoscopic and non-bronchoscopic investigations. This review will take necessary precaution when interpreting subgroup analysis for studies reporting unspecific microbiological confirmation. An a priori subgroup analysis by method of diagnosis is planned according to the following categories: blinded outcome assessment with adjudication, unblinded outcome assessment with adjudication, blinded outcome assessment without adjudication, and unblinded outcome assessment without adjudication.
- Presence and type of immune suppression. This may refer to patients who are immunocompromised, as patients with human immunodeficiency virus (HIV) infection or acquired immune deficiency syndrome (AIDS), oncologic conditions, or history of immunosuppressant drug use have increased risks of developing VAP and clinical failure (e.g., death) following institution of invasive mechanical ventilation. An a priori subgroup analysis by presence (or absence) and type of immune suppression is planned for the following categories: according to presence (or absence), immunocompromised and non-immunocompromised; according to type, deliberately induced (by immunosuppressive medications) and non-deliberately induced immunosuppression (by many types of cancers, HIV/AIDS).
- Presence and type of initial antimicrobial therapy. This may refer to patients who received empirical antimicrobial treatment(s). The term “empirical” is defined as treatment given prior to obtaining susceptibility results of microbial analysis. An a priori subgroup analysis by presence (or absence) and type of initial antimicrobial therapy is planned for the following categories: according to presence (or absence), with initial antimicrobial therapy and without initial antimicrobial therapy; according to type, appropriate and inappropriate empiric antibiotics.
- Presence and type of VAP protocol. This may refer to studies reporting the presence or absence of a VAP prevention protocol, as the number of VAP prevention strategies (e.g., VAP bundles) that are routinely available may influence VAP occurrence. An a priori subgroup analysis by presence (or absence) and type of VAP protocol is planned for the following categories: according to presence (or absence), units with VAP prevention strategies and units without VAP prevention strategies; according to types, recommended VAP bundles and modified VAP bundles.

The abovementioned subgroup analyses will be formally applied to all outcome measures even if the variability appears to be explained by chance. If studies yield large inconsistent results due to serious or unexplained heterogeneity, the review investigators plan to rate down the quality of evidence for each outcome measure using the recommended approach [32].

Furthermore, this present review anticipates that all these proposed a priori subgroup analyses might not be adequately reported in the included studies. Hence, additional caution will be regarded when exploring and interpreting the review results, as underpowered subgroup analysis, in full awareness of the review investigators, may lead to an incorrect conclusion.

#### Sensitivity analysis

A sensitivity analysis will be performed in studies with low risk of bias to ensure the consistency of pooled results concerning the association between variables. This approach involves the omission of outliers or reports with a higher risk of bias, indicating less rigorous designs. These may include studies with methodological and statistical issues (i.e., studies reporting composite outcomes), non-equivalent controls (i.e., studies not reporting the minimum duration of MV in non-VAP patients), inconsistent VAP definition or questionable diagnostic criteria for VAP, and studies that did not report/assess the presence lung infection at admission. Pooled results with 95% CIs will again be analyzed to test for the robustness of association. The conclusion would be regarded as stable if the results do not change substantially, indicating a higher degree of certainty. The comparison of original risk estimates with post-assessment results will be displayed in a summary table.

#### Assessment of reporting biases

If there are at least ten relevant studies with robust designs, a visual asymmetry assessment using funnel plot will be generated to detect publication bias [33].

### Confidence in cumulative evidence

The present review will use the Grading of Recommendations Assessment, Development, and Evaluation (GRADE) approach [34] to assess the confidence in estimates of overall prognosis. In this approach, the process used for critical appraisal (i.e., evaluation by 2 reviewers with consensus or a third-party adjudication) in risk of bias assessment will be applied, considering study limitations (risk of bias), imprecision, inconsistency, indirectness, and publication bias. The confidence in cumulative evidence for each epidemiological index or outcome measure will be graded in one of four levels: high, moderate, low, and very low. A summary of findings using GRADEpro GDT software will be generated to illustrate the confidence in cumulative evidence in all outcome measures [35].

## Discussion

Ventilator-associated pneumonia is a complex and multifactorial clinical condition associated with high morbidity and mortality and has a staggering impact on healthcare costs. This premise is largely recognized in the international body of literature [36] with little disagreement [37, 38]. However, the clinical epidemiology and outcomes of VAP in broad subgroups of critically ill adult populations have not been clearly defined and much remains to be understood. To date, a large number of primary studies have been identified; thus far, none are conclusive. The present study is the first review and meta-analysis that aims to investigate the clinical epidemiology of VAP, including the overall prognosis of and risk factors for morbidity and mortality outcomes associated with this condition in greater detail.

The relevance of this present review highlights the urgent need to identify medium- and high-risk critically ill patients targeting important risk factors for modification and stratified management. The review results may be used as a springboard for tool development (i.e., VAP risk index) and clinical protocol design, which raises a number of critical issues for research, policy, and clinical practice considerations. Moreover, the review results are critical to shed light on other potential predictors of VAP morbidity and mortality outcomes, warranting further investigation.

This present review, of course, has inherent strengths and limitations. Strengths include an exhaustive search of evidence, published or unpublished, without date and language restrictions in five major scientific databases and other sources; a comprehensive risk analysis plan with a justified number of a priori subgroup analyses (including a priori hypotheses with a pre-specified direction); and the utilization of the GRADE approach to rate the quality of evidence. Limitations relate to the anticipated variability in critically ill populations, diagnostic methods used, and study designs reported from included studies; paucity of data regarding the clinical epidemiology and outcomes of VAP in some countries; and the lack of subgroup and adjusted analyses of prognostic factors within-study comparisons, as no individual patient data will be obtained.

With this initiative effort to provide a substantial link for the successful implementation of the VAP prevention practices in critical care settings, the key players such as healthcare practitioners, researchers, policymakers, and hospital administrators are hoped to make productive use of the review results, which is soon to be disseminated following this protocol.

## Additional files

Additional file 1: Preferred Reporting Items for Systematic review and Meta-Analysis for Protocols (PRISMA-P) 2015 guidelines. (DOCX 44 kb)

Additional file 2: Supplementary tables. (DOCX 132 kb)

Additional file 3: Flow diagrams. (DOCX 402 kb)

Additional file 4: Data forms. (DOCX 161 kb)

## Data Availability

Not applicable.

## Abbreviations

CENTRAL: The Cochrane Central Register of Controlled Trials
CI: Confidence interval
CINAHL: Cumulative Index to Nursing and Allied Health Literature
CPIS: Clinical pulmonary infection score
EMBASE: Excerpta Medica dataBASE
ETT: Endotracheal tube
GRADE: Grading of Recommendations Assessment, Development, and Evaluation
ICU: Intensive care unit
IV: Inverse variance
LOS: Length of stay
MH: Mantel-Haenszel
MV: Mechanical ventilation
NLM: National Library of Medicine
OR: Odds ratio
PI: Principal investigator
PICO: Patients, interventions, comparators, outcomes
PRISMA-P: Preferred Reporting Items for Systematic review and Meta-Analysis Protocols
PROSPERO: International Prospective Register of Systematic Reviews
QUIPS: Quality In Prognosis Studies
RA: Research assistant
RCT: Randomized controlled trials
RevMan: Review Manager
VAE: Ventilator-associated event
VAP: Ventilator-associated pneumonia

## References

[1] Divatia JV, Bhowmick K (2005). Complications of endotracheal intubation and other airway management procedures. Indian J Anaesth..

[2] Mutlu GM, Factor P (2000). Complications of mechanical ventilation. Respir Care Clin N Am..

[3] Dosemeci L, Yilmaz M, Celikbilek G, Yagmur R, Ramazanoglu A (2003). The complications associated with mechanical ventilation. Crit Care..

[4] Rubano JA, Paccione MF, Rutigliano DN (2015). Outcomes following prolonged mechanical ventilation: analysis of a countywide trauma registry. J Trauma Acute Care Surg..

[5] Richards MJ, Edwards JR, Culver DH, Gaynes RP (1999). Nosocomial infections in medical intensive care units in the United States. National Nosocomial Infections Surveillance System. Crit Care Med.

[6] Tablan OC, Anderson LJ, Besser R, Bridges C, Hajjeh R, CDC; Healthcare Infection Control Practices Advisory Committee (2004). Guidelines for preventing health-care--associated pneumonia, 2003: recommendations of CDC and the Healthcare Infection Control Practices Advisory Committee. MMWR Recomm Rep..

[7] Smyth ET, McIlvenny G, Enstone JE (2008). Four country healthcare associated infection prevalence survey 2006: overview of the results. J Hosp Infect..

[8] Humphreys H, Newcombe RG, Enstone J (2010). Four country healthcare-associated infection prevalence survey: pneumonia and lower respiratory tract infections. J Hosp Infect..

[9] American Thoracic Society, Infectious Diseases Society of America (2005). Guidelines for the management of adults with hospital acquired, ventilator-associated, and healthcare-associated pneumonia. Am J Respir Crit Care Med..

[10] Coppadoro A, Bittner E, Berra L (2012). Novel preventive strategies for ventilator-associated pneumonia. Crit Care..

[11] Nseir S, Ader F, Lubret R, Marquette CH (2011). Pathophysiology of airway colonization in critically ill COPD patient. Curr Drug Targets..

[12] Chastre J, Fagon JY (2002). Ventilator-associated pneumonia. Am J Respir Crit Care Med..

[13] Bonten MJ, Kollef MH, Hall JB (2004). Risk factors for ventilator-associated pneumonia: from epidemiology to patient management. Clin Infect Dis..

[14] Arabi Y, Al-Shirawi N, Memish Z, Anzueto A (2008). Ventilator-associated pneumonia in adults in developing countries: a systematic review. Int J Infect Dis..

[15] Tan B, Zhang F, Zhang X (2014). Risk factors for ventilator-associated pneumonia in the neonatal intensive care unit: a meta-analysis of observational studies. Eur J Pediatr..

[16] Liu B, Li SQ, Zhang SM (2013). Risk factors of ventilator-associated pneumonia in pediatric intensive care unit: a systematic review and meta-analysis. J Thorac Dis..

[17] Fitch ZW, Whitman GJ (2014). Incidence, risk, and prevention of ventilator-associated pneumonia in adult cardiac surgical patients: a systematic review. J Card Surg..

[18] He S, Chen B, Li W (2014). Ventilator-associated pneumonia after cardiac surgery: a meta-analysis and systematic review. J Thorac Cardiovasc Surg..

[19] Siempos II, Athanassa Z, Falagas ME (2008). Frequency and predictors of ventilator-associated pneumonia recurrence: a meta-analysis. Shock..

[20] Moola S, Munn Z, Sears K (2015). Conducting systematic reviews of association (etiology): the Joanna Briggs Institute’s approach. Int J Evid Based Healthc.

[21] Moher D, Liberati A, Tetzlaff J (2009). Preferred reporting items for systematic reviews and meta-analyses: the PRISMA statement. PLoS Med..

[22] Shamseer L, Moher D, Clarke M (2015). Preferred reporting items for systematic review and meta-analysis protocols (PRISMA-P) 2015: elaboration and explanation. BMJ..

[23] World Bank. World development indicators 2011. World Development Indicators, Washington, DC. 2011. http://documents.worldbank.org/curated/en/245401468331253857/World-development-indicators-2011. Accessed 28 May 2017.

[24] Wunderink R (2000). Clinical criteria in the diagnosis of ventilator-associated pneumonia. Chest..

[25] Magill SS, Klompas M, Balk R (2013). Developing a new, national approach to surveillance for ventilator-associated events: executive summary. Infect Control Hosp Epidemiol..

[26] Hayden JA, van der Windt DA, Cartwright JL, Côté P, Bombardier C (2013). Assessing bias in studies of prognostic factors. Ann Intern Med..

[27] Heyland DK, Cook DJ, Griffith L, Keenan SP, Brun-Buisson C (1999). The attributable morbidity and mortality of ventilator-associated pneumonia in the critically ill patient. The Canadian Critical Trials Group. Am J Respir Crit Care Med.

[28] Landis JR, Koch GG (1977). The measurement of observer agreement for categorical data. Biometrics.

[29] Krueger WA, Lenhart FP, Neeser G (2002). Influence of combined intravenous and topical antibiotic prophylaxis on the incidence of infections, organ dysfunctions, and mortality in critically ill surgical patients: a prospective, stratified, randomized, double-blind, placebo-controlled clinical trial. Am J Respir Crit Care Med..

[30] Nguile-Makao M, Zahar JR, Français (2010). Attributable mortality of ventilator-associated pneumonia: respective impact of main characteristics at ICU admission and VAP onset using conditional logistic regression and multi-state models. Intensive Care Med..

[31] United Nations Conference on Trade and Development. Development status groups and composition. 2018. https://unctadstat.unctad.org/en/Classifications/DimCountries_DevelopmentStatus_Hierarchy.pdf. Accessed 18 Feb 2019.

[32] Guyatt G, Oxman A, Kunz R (2011). GRADE guidelines: 7. Rating the quality of evidence—inconsistency. J Clin Epidemiol..

[33] Egger M, Smith GD, Phillips AN (1997). Meta-analysis: principles and procedures. BMJ..

[34] Balshem H, Helfand M, Schünemann HJ (2011). GRADE guidelines: 3. Rating the quality of evidence. J Clin Epidemiol..

[35] GARDEpro GDT. GRADEpro Guideline Development Tool [Software]. McMaster University, Hamilton (developed by Evidence Prime, Inc.). 2015. https://gradepro.org/cite/. Accessed 18 Oct 2018.

[36] Safdar N, Dezfulian C, Collard HR, Saint S (2005). Clinical and economic consequences of ventilator-associated pneumonia: a systematic review. Crit Care Med..

[37] Magret M, Amaya-Villar R, Garnacho J (2010). Ventilator-associated pneumonia in trauma patients is associated with lower mortality: results from EU-VAP study. J Trauma..

[38] Siempos II, Vardakas KZ, Kyriakopoulos CE, Ntaidou TK, Falagas ME (2010). Predictors of mortality in adult patients with ventilator-associated pneumonia: a meta-analysis. Shock..

