# Supplementary material for: Clinical epidemiology and outcomes of ventilator-associated pneumonia in critically ill adult patients: protocol for a large-scale systematic review and planned meta-analysis
Source: Syst Rev. 2019 Jul 20;8:180. doi: 10.1186/s13643-019-1080-y (PMC6642735; doi:10.1186/s13643-019-1080-y)
Supplement: Supplementary file 2 — Supplementary tables. (DOCX 132 kb) [file 13643_2019_1080_MOESM2_ESM.docx]

| **Table S1** MEDLINE (via PubMed) search strategy |
| --- |
| #1, Adult or mechanically ventilated  #2, Critically ill or intensive care or critical care or ICU  #3, #1 OR #2  #4, Independent risk factor* or risk factor* or risk or predictor* or exposure or mechanical ventilation or intubation  #5, VAP group or non-VAP group or with VAP or without VAP  #6, Pneumonia, ventilator-associated or ventilator-associated pneumonia or ventilator associated pneumonia or ventilator-associated or ventilator-acquired or VAP or early VAP or late VAP or nosocomial pneumonia  #7, Incidence or prevalence or VAP rate  #8, Length of stay or LOS or duration of mechanical ventilation or intubation  #9, Antibiotic cost or hospital* cost  #10, Morbidity or mortality or fatal* or death or attributable mortality or mortality rate or case fatality rate  #11, #6 OR #7 OR #8 OR #9 OR #10  #12, Randomized controlled trial or RCT or randomised control trial or clinical trial or experimental stud* or cohort stud* or case-control stud* or observational stud* or prospective analysis or prospective cohort or retrospective analysis or retrospective cohort  #13, #3 AND #4 AND #5 AND #11 AND #12 |
| Combined search results (PECOS): 1,095 citations (as of 2 May 2018) |

| **Table S2** CENTRAL (via Cochrane Library) search strategy |
| --- |
| #1 Mesh descriptor Adult explode all trees  #2 (critical* in All Text near/6 ill* in All Text)  #3 (intensive in All Text near/6 patient* in All Text)  #4 MeSH descriptor Critical care this term only  #5 (intensive-care in All Text or “intensive care” in All Text or critical-care in All Text or “critical care” in All Text)  #6 ICU in Title, Abstract or Keywords  #7 ((intubat* in All Text near/5 patient* in All Text) or (ventilat* in All Text near/5 patient* in All Text))  #8 (#1 or #2 or #3 or #4 or #5 or #6 or #7)  #9  “Risk factor” in Tile, Abstract or Keywords  #10 “risk factor” in All Text  #11 (predictor* in All text or exposure* in All Text)  #12 (#9 or #10 or #11)  #13 (VAP group in All Text or non-VAP group in All text)  #14 (with VAP in All Text or without VAP in All Text)  #15 (survivor in All Text or non-survivor in All Text)  #16 (#13 or #14 or #15)  #17 MeSH descriptor Pneumonia, ventilator-associated explode all trees  #18 (VAP in Title, Abstract or Keywords or “ventilator-associated pneumonia” in Title, Abstract or Keywords)  #19 “VAP” in Title, Abstract or Keywords  #20 “nosocomial infection*” in Title, Abstract or Keywords  #21 “nosocomial pneumonia” in Title, Abstract or Keywords  #22 (“ventilator-acquired pneumonia” in Title, Abstract or Keywords  or ventilator acquired pneumonia” in Title, Abstract or Keywords)  #23 early VAP in All Text  #24 late VAP in All Text  #25 incidence in All Text  #26 prevalence in All Text  #27 duration of mechanical ventilation in All Text  #28 length of stay in All Text  #29 (hospitalization cost in All Text  or hospital cost in All Text)  #30 antibiotic cost in All Text  #31 (#17 or #18 or #19 or #20 or #21 or #22 or #23 or #24 or #25 or #26 or #27 or #28 or #29 or #30)  #32  (“Randomized controlled trial” in All Text or “randomised control trial” in All Text or “RCT” in All Text or “clinical trial” in All Text or “experimental stud*” in All Text or “cohort stud*” in All Text or “case-control stud*” in All Text or observational stud* in All Text or “prospective analysis” in All Text or “prospective cohort” in All Text or “retrospective analysis” in All Text or “retrospective cohort” in All Text)  #33 (Randomized controlled trial in Title, Abstract or Keywords or randomized clinical trial in Title, Abstract or Keywords or randomised control trial in Title, Abstract or Keywords)  #34 (Cohort study in Title, Abstract or Keywords or case-control study in Title, Abstract or Keywords)  #35 (Retrospective in Title, Abstract or Keywords or Prospective in Title, Abstract or Keywords)  #36 (#32 or #33 or #34 or #35)  #37 (#8 and #12 and #16 and #31 and #36) |
| Combined search results (PECOS): 53 citations (as of 13 March 2017) |

| **Table S3** CINAHL (via EBSCO*host*) search strategy |
| --- |
| S1, Adult or mechanically ventilated  S2, Critically ill or intensive care or critical care or ICU  S3, S1 OR S2  S4, Independent risk factor* or risk factor* or risk or predictor* or exposure or mechanical ventilation or intubation  S5, VAP group or non-VAP group or with VAP or without VAP  S6, Pneumonia, ventilator-associated or ventilator-associated pneumonia or ventilator associated pneumonia or ventilator-associated or ventilator-acquired or VAP or early VAP or late VAP or nosocomial pneumonia  S7, Incidence or prevalence or VAP rate  S8, Length of stay or LOS or duration of mechanical ventilation or intubation  S9, Antibiotic cost or hospital* cost  S10, Morbidity or mortality or fatal* or death or attributable mortality or mortality rate or case fatality rate  S11, S6 OR S7 OR S8 OR S9 OR S10  S12, Randomized controlled trial or RCT or randomised control trial or clinical trial or experimental stud* or cohort stud* or case-control stud* or observational stud* or prospective analysis or prospective cohort or retrospective analysis or retrospective cohort  S13, S3 AND S4 AND S5 AND S11 AND S12 |
| Combined search results (PECOS): 289 citations (as of 13 March 2017) |

| **Table S4** Web of Science (via Saudi Digital Library) search strategy |
| --- |
| #1, TS=(Adult or mechanically ventilated)  #2, TS=(Critically ill or intensive care or critical care or ICU)  #3, #1 OR #2  #4, TS=(Independent risk factor* or risk factor* or risk or predictor* or exposure or mechanical ventilation or intubation)  #5, TS=(VAP group or non-VAP group or with VAP or without VAP)  #6, TS=(Pneumonia, ventilator-associated or ventilator-associated pneumonia or ventilator associated pneumonia or ventilator-associated or ventilator-acquired or VAP or early VAP or late VAP or nosocomial pneumonia)  #7, TS=(Incidence or prevalence or VAP rate)  #8, TS=(Length of stay or LOS or duration of mechanical ventilation or intubation)  #9, TS=(Antibiotic cost or hospital* cost)  #10, TS=(Morbidity or mortality or fatal* or death or attributable mortality or mortality rate or case fatality rate)  #11, #6 OR #7 OR #8 OR #9 OR #10  #12, TS=(Randomized controlled trial or RCT or randomised control trial or clinical trial or experimental stud* or cohort stud* or case-control stud* or observational stud* or prospective analysis or prospective cohort or retrospective analysis or retrospective cohort)  #13, #3 AND #4 AND #5 AND #11 AND #12 |
| Combined search results (PECOS): 604 citations (as of 13 March 2017 |

| **Table S5** EMBASE search strategy |
| --- |
| #1, adult:ti,ab,kw OR 'ventilated patient':ti,ab,kw  #2, 'critically ill':ti,ab,kw OR 'intensive care':ti,ab,kw OR 'critical care':ti,ab,kw OR icu:ti,ab,kw  #3, #1 OR #2  #4, 'independent risk factor*':ti,ab,kw OR 'risk factor*':ti,ab,kw OR risk:ti,ab,kw OR predictor*:ti,ab,kw OR exposure:ti,ab,kw OR 'mechanical ventilation':ti,ab,kw OR intubation:ti,ab,kw  #5, 'vap group':ti,ab,kw OR 'non-vap group':ti,ab,kw OR 'with vap':ti,ab,kw OR 'without vap':ti,ab,kw  #6, 'pneumonia, ventilator-associated':ti,ab,kw OR 'ventilator-associated pneumonia':ti,ab,kw OR 'ventilator associated pneumonia':ti,ab,kw OR 'ventilator associated':ti,ab,kw OR 'ventilator acquired':ti,ab,kw OR vap:ti,ab,kw OR 'early vap':ti,ab,kw OR 'late vap':ti,ab,kw OR 'nosocomial pneumonia':ti,ab,kw  #7, incidence:ti,ab,kw OR prevalence:ti,ab,kw OR 'vap rate':ti,ab,kw  #8, 'length of stay':ti,ab,kw OR los:ti,ab,kw OR 'duration of mechanical ventilation':ti,ab,kw OR intubation:ti,ab,kw  #9, 'antibiotic cost':ti,ab,kw OR 'hospital* cost':ti,ab,kw  #10, morbidity:ti,ab,kw OR mortality:ti,ab,kw OR fatal*:ti,ab,kw OR death:ti,ab,kw OR 'attributable mortality':ti,ab,kw OR 'mortality rate':ti,ab,kw OR 'case fatality rate':ti,ab,kw  #11, #6 OR #7 OR #8 OR #9 OR #10  #12, 'randomized controlled trial':ti,ab,kw OR rct:ti,ab,kw OR 'randomised control trial':ti,ab,kw OR 'clinical trial':ti,ab,kw OR 'experimental stud*':ti,ab,kw OR 'cohort stud*':ti,ab,kw OR 'case-control stud*':ti,ab,kw OR 'observational stud*':ti,ab,kw OR 'prospective analysis':ti,ab,kw OR 'prospective cohort':ti,ab,kw OR 'retrospective analysis':ti,ab,kw OR 'retrospective cohort':ti,ab,kw  #13, #3 AND #4 AND #5 AND #11 AND #12 |
| Combined search results (PECOS): 200 citations (as of 18 May 2019) |

| **Table S6** Lists of relevant medical and nursing Journals selected for journal hand search* | | | | |
| --- | --- | --- | --- | --- |
| Medical journals | |  | Nursing journals | |
| NLM title abbreviation | Publication start year |  | NLM title abbreviation | Publication start year |
| *Am J Epidemiol*  *Am J Infect Control*  *Am J Med*  *Am J Respir Crit Care Med*  *Anaesth Intensive Care*  *Ann Intensive Care*  *Ann Intern Med*  *Ann Thorac Cardiovasc Surg*  *Ann Thorac Med*  *Ann Thorac Surg*  *BMC Infect Dis*  *BMC Pulmo Med*  *Braz J Infect Dis*  *Can J Infect Dis Med Microbiol*  *Can Respir J*  *Chest*  *Clin Infect Dis*  *Clin Microbiol Infect*  *Clin Respir J*  *Comp Immunol Microbiol Infect Dis*  *Crit Care*  *Crit Care Clin*  *Crit Care Med*  *Curr Infect Dis Rep*  *Curr Opin Crit Care*  *Curr Opin Infect Dis*  *Curr Opin Pulm Med*  *Diagn Microbiol Infect Dis*  *Emerg Infect Dis*  *Emerg Microbes Infect*  *Epidemiol Infect*  *Eur J Clin Microbiol Infect Dis*  *Euro J Respir Dis*  *Eur Respir J*  *Expert Rev Respir Med*  *Heart Lung*  *Indian J Crit Care Med*  *Infection*  *Infect Control Hosp Epidemiol*  *Infect Dis Clin North Am*  *Intensive Care Med*  *Int J Infect Dis*  *JAMA*  *Jpn J Infect Dis*  *J Cardiothorac Vasc Anesth*  *J Clin Epidmeiol*  *J Crit Care*  *J Hosp Infect*  *J infect*  *J Infect Chemother*  *J Infect Public Health*  *J Infect Dev Ctries*  *J Infect Dis*  *J Intensive Care Med*  *J Microbiol Immunol Infect*  *J Neurosurg Anesthesiol*  *J Thorac Cardiovasc Surg*  *J Trauma Acute Care Surg*  *Lancet Infect Dis*  *Lancet Respir Med*  *Lung India*  *Minerva Anestesiol*  *Neurocrit Care*  *N Engl J Med*  *Respir Care*  *Respir Res*  *Scand J Infect Dis*  *Scand J Respir Dis*  *Semin Respir Crit Care Med*  *Surg Infect (Larchmt)*  *Transpl Infect Dis* | 1965  1980  1946  1994  1972  2011  1927  1995  2006  1965  2001  2001  1997  2004  1994  1970  1992  1995  2007  1997  1978  1985  1973  1999  1995  1988  1995  1983  1995  2012  1987  1988  1980  1988  2007  1972  1997  1973  1988  1987  1977  1996  1960  1999  1991  1988  1986  1980  1979  1995  2008  2007  1904  1986  1998  1989  1959  2012  2001  2013  1982  1953  2004  1928  1971  2000  1969  1966  1994  2001  1999 |  | *Am J Crit Care*  *Aust Crit Care*  *Crit Care Nurse*  *Crit Care Nurs Clin North Am*  *Dimens Crit Care Nurs*  *Intensive Crit Care Nurs*  *J Neurosci Nurs*  *J Trauma Nurs*  *Nurs Crit Care* | 1992  1992  1980  1989  1982  1992  1986  1994  1996 |
| NLM, National Library Medicine.  *Search strategy: “<journal NLM title abbreviation>”[jour] AND ventilator-associated pneumonia | | | | |

| **Table S7** Summary of research questions and sample planned data analyses | | |
| --- | --- | --- |
| **Research question** | **Analysis type** | **Planned data analysis** |
| 1. What is the clinical profile of VAP and non-VAP patients? | Descriptive statistics | Analysis 1. Number or frequency/proportion, percentage, mean (±SD) or median (IQR) and Chi-squared test |
| 1. What is the prevalence of VAP in critically ill adult patients? | Descriptive epidemiology | Analysis 2. Prevalence rate (per 10,000 population or per 100,000 population) |
| 1. What pathogens are commonly associated in patients with VAP? | Descriptive statistics | Analysis 3. Number or frequency, percentage, mean (±SD) |
| 1. Is there a significant difference in the VAP causative pathogens among survivors and non-survivors/patients with EOVAP and LOVAP? | Inferential statistics | Analysis 4. Comparison of two proportions |
| 1. What factors may increase the risk of ventilator-associated pneumonia and are associated with poor clinical outcomes (VAP-associated mortality, longer duration of MV, increased LOS in ICU/hospital, microbial colonization, antibiotic and hospitalization costs) in critically ill adult patients, in terms of:    1. Host- or patient-related factors;    2. Treatment- or intervention-related factors;    3. Personnel-related factors;    4. Device-related factors;    5. Environmental factors;    6. Other related factors? | Meta-analyses (Risk factor analyses) | **Ventilator-associated pneumonia is multifactorial in nature. Therefore, asking specific questions relative to risk factors cover a wide range of clinical data. To analyze these factors, the investigator asked “general” questions related to six VAP factors. Specific clinical research questions for all mentioned factors will be provided following data extraction and management. The following are examples of specific clinical research questions that might be asked according to the following related factors:**  **Host- or patient-related factors: *“Do patients with advanced age influence VAP development?”***  Example Analysis: Comparison 1 Advanced age observed in cases and controls, Outcome 1. Incidence of VAP  **Treatment or intervention-related factors:  *“Do re-intubation procedures influence VAP development?”***  Example Analysis: Comparison 2 Observed re-intubation procedures in cases and controls, Outcome 1. Incidence of VAP  **Personnel-related factors:  *“Do understaffing issues influence VAP development?”***  Example Analysis: Comparison 3 Understaffing exposures of cases and controls, Outcome 1. Incidence of VAP  **Device-related factors:  *“Do routine ventilator circuit changes influence VAP development?”***  Example Analysis: Comparison 4 Routine ventilator circuit changes observed in cases and controls, Outcome 1. Incidence of VAP  **Environmental-related factors:  *“Does fall season influence VAP development?”***  Example Analysis: Comparison 5 Fall season exposures of cases and controls, Outcome 1. Incidence of VAP  **Other related factors:  *“Do desaturation episodes influence VAP development?”***  Example Analysis: Comparison 6 Desaturation episodes in cases and controls, Outcome 1. Incidence of VAP  Analysis 5. Risk factor analyses:  **5.1. Host- or patient-related factors.**  Analysis 5.1.1. Comparison 1. Host- or patient-related risk factors among cases and controls, Outcome 1. Incidence of VAP (Decreased risk or increased risk?)  Analysis 5.1.2. Comparison 1. Host- or patient-related risk factors among cases and controls, Outcome 3. ICU mortality associated with VAP (Decreased risk or increased risk?)  Analysis 5.1.3. Comparison 1. Host- or patient-related risk factors among cases and controls, Outcome 4. In-hospital mortality associated with VAP (Decreased risk or increased risk?)  Analysis 5.1.4. Comparison 1. Host- or patient-related risk factors among cases and controls, Outcome 5. Duration of MV (Decreased risk or increased risk?)  Analysis 5.1.5. Comparison 1. Host- or patient-related risk factors among cases and controls, Outcome 6. ICU LOS (Decreased risk or increased risk?)  Analysis 5.1.6. Comparison 1. Host- or patient-related risk factors among cases and controls, Outcome 7. Hospital LOS (Decreased risk or increased risk?)  Analysis 5.1.7. Comparison 1. Host- or patient-related risk factors among cases and controls, Outcome 8. Microbial colonization (Decreased risk or increased risk?)  Analysis 5.1.8. Comparison 1. Host- or patient-related risk factors among cases and controls, Outcome 9. Cost of antibiotic treatment of VAP (Decreased risk or increased risk?) |

| **Table S7** Continued | | |
| --- | --- | --- |
| **Research question** | **Analysis type** | **Planned data analysis** |
|  |  | Analysis 5.1.9. Comparison 1. Host- or patient-related risk factors among cases and controls, Outcome 10. Hospitalization cost (Decreased risk or increased risk?)  **5.2. Treatment- or intervention-related factors.**  Analysis 5.2.1. Comparison 2. Treatment- or intervention-related risk factors among cases and controls, Outcome 1. Incidence of VAP (Decreased risk or increased risk?)  Analysis 5.2.2. Comparison 2. Treatment- or intervention-related risk factors among cases and controls, Outcome 3. ICU mortality associated with VAP (Decreased risk or increased risk?)  Analysis 5.2.3. Comparison 2. Treatment- or intervention-related risk factors among cases and controls, Outcome 4. In-hospital mortality associated with VAP (Decreased risk or increased risk?)  Analysis 5.2.4. Comparison 2. Treatment- or intervention-related risk factors among cases and controls, Outcome 5. Duration of MV (Decreased risk or increased risk?)  Analysis 5.2.5. Comparison 2. Treatment- or intervention-related risk factors among cases and controls, Outcome 6. ICU LOS (Decreased risk or increased risk?)  Analysis 5.2.6. Comparison 2. Treatment- or intervention-related risk factors among cases and controls, Outcome 7. Hospital LOS (Decreased risk or increased risk?)  Analysis 5.2.7. Comparison 2. Treatment- or intervention-related risk factors among cases and controls, Outcome 8. Microbial colonization (Decreased risk or increased risk?)  Analysis 5.2.8. Comparison 2. Treatment- or intervention-related risk factors among cases and controls, Outcome 9. Cost of antibiotic treatment of VAP (Decreased risk or increased risk?)  Analysis 5.2.9. Comparison 2. Treatment- or intervention-related risk factors among cases and controls, Outcome 10. Hospitalization cost (Decreased risk or increased risk?)  **5.3. Personnel-related factors.**  Analysis 5.3.1. Comparison 3. Personnel-related risk factors among cases and controls, Outcome 1. Incidence of VAP (Decreased risk or increased risk?)  Analysis 5.3.2. Comparison 3. Personnel-related risk factors among cases and controls, Outcome 3. ICU mortality associated with VAP (Decreased risk or increased risk?)  Analysis 5.3.3. Comparison 3. Personnel-related risk factors among cases and controls, Outcome 4. In-hospital mortality associated with VAP (Decreased risk or increased risk?)  Analysis 5.3.4. Comparison 3. Personnel-related risk factors among cases and controls, Outcome 5. Duration of MV (Decreased risk or increased risk?)  Analysis 5.3.5. Comparison 3. Personnel-related risk factors among cases and controls, Outcome 6. ICU LOS (Decreased risk or increased risk?)  Analysis 5.3.6. Comparison 3. Personnel-related risk factors among cases and controls, Outcome 7. Hospital LOS (Decreased risk or increased risk?)  Analysis 5.3.7. Comparison 3. Personnel-related risk factors among cases and controls, Outcome 8. Microbial colonization (Decreased risk or increased risk?)  Analysis 5.3.8. Comparison 3. Personnel-related risk factors among cases and controls, Outcome 9. Cost of antibiotic treatment of VAP (Decreased risk or increased risk?)  Analysis 5.3.9. Comparison 3. Personnel-related risk factors among cases and controls, Outcome 10. Hospitalization cost (Decreased risk or increased risk?)  **5.4. Device-related factors.**  Analysis 5.4.1. Comparison 4. Device-related risk factors among cases and controls, Outcome 1. Incidence of VAP (Decreased risk or increased risk?)  Analysis 5.4.2. Comparison 4. Device-related risk factors among cases and controls, Outcome 3. ICU mortality associated with VAP (Decreased risk or increased risk?)  Analysis 5.4.3. Comparison 4. Device-related risk factors among cases and controls, Outcome 4. In-hospital mortality associated with VAP (Decreased risk or increased risk?)  Analysis 5.4.4. Comparison 4. Device-related risk factors among cases and controls, Outcome 5. Duration of MV (Decreased risk or increased risk?)  Analysis 5.4.5. Comparison 4. Device-related risk factors among cases and controls, Outcome 6. ICU LOS (Decreased risk or increased risk?) |

| **Table S7** Continued | | |
| --- | --- | --- |
| **Research question** | **Analysis type** | **Planned data analysis** |
|  |  | Analysis 5.4.6. Comparison 4. Device-related risk factors among cases and controls, Outcome 7. Hospital LOS (Decreased risk or increased risk?)  Analysis 5.4.7. Comparison 4. Device-related risk factors among cases and controls, Outcome 8. Microbial colonization (Decreased risk or increased risk?)  Analysis 5.4.8. Comparison 4. Device-related risk factors among cases and controls, Outcome 9. Cost of antibiotic treatment of VAP (Decreased risk or increased risk?)  Analysis 5.4.9. Comparison 4. Device-related risk factors among cases and controls, Outcome 10. Hospitalization cost (Decreased risk or increased risk?)  **5.5. Environmental-related factors.**  Analysis 5.5.1. Comparison 5. Environmental-related risk factors among cases and controls, Outcome 1. Incidence of VAP (Decreased risk or increased risk?)  Analysis 5.5.2. Comparison 5. Environmental-related risk factors among cases and controls, Outcome 3. ICU mortality associated with VAP (Decreased risk or increased risk?)  Analysis 5.5.3. Comparison 5. Environmental-related risk factors among cases and controls, Outcome 4. In-hospital mortality associated with VAP (Decreased risk or increased risk?)  Analysis 5.5.4. Comparison 5. Environmental-related risk factors among cases and controls, Outcome 5. Duration of MV (Decreased risk or increased risk?)  Analysis 5.5.5. Comparison 5. Environmental-related risk factors among cases and controls, Outcome 6. ICU LOS (Decreased risk or increased risk?)  Analysis 5.5.6. Comparison 5. Environmental-related risk factors among cases and controls, Outcome 7. Hospital LOS (Decreased risk or increased risk?)  Analysis 5.5.7. Comparison 5. Environmental-related risk factors among cases and controls, Outcome 8. Microbial colonization (Decreased risk or increased risk?)  Analysis 5.5.8. Comparison 5. Environmental-related risk factors among cases and controls, Outcome 9. Cost of antibiotic treatment of VAP (Decreased risk or increased risk?)  Analysis 5.5.9. Comparison 5. Environmental-related risk factors among cases and controls, Outcome 10. Hospitalization cost (Decreased risk or increased risk?)  **5.6. Other related factors.**  Analysis 5.6.1. Comparison 6. Other related risk factors among cases and controls, Outcome 1. Incidence of VAP (Decreased risk or increased risk?)  Analysis 5.6.2. Comparison 6. Other related risk factors among cases and controls, Outcome 3. ICU mortality associated with VAP (Decreased risk or increased risk?)  Analysis 5.6.3. Comparison 6. Other related risk factors among cases and controls, Outcome 4. In-hospital mortality associated with VAP (Decreased risk or increased risk?)  Analysis 5.6.4. Comparison 6. Other related risk factors among cases and controls, Outcome 5. Duration of MV (Decreased risk or increased risk?)  Analysis 5.6.5. Comparison 6. Other related risk factors among cases and controls, Outcome 6. ICU LOS (Decreased risk or increased risk?)  Analysis 5.6.6. Comparison 6. Other related risk factors among cases and controls, Outcome 7. Hospital LOS (Decreased risk or increased risk?)  Analysis 5.6.7. Comparison 6. Other related risk factors among cases and controls, Outcome 8. Microbial colonization (Decreased risk or increased risk?)  Analysis 5.6.8. Comparison 6. Other related risk factors among cases and controls, Outcome 9. Cost of antibiotic treatment of VAP (Decreased risk or increased risk?)  Analysis 5.6.9. Comparison 6. Other related risk factors among cases and controls, Outcome 10. Hospitalization cost (Decreased risk or increased risk?) |
| 6. What is the proportional attributable mortality of VAP? | Descriptive epidemiology | Analysis 6. Proportional crude mortality (%) reported as RRI |
| 7. Can VAP be attributed to patient mortality?  7.1. VAP as predictor of patient mortality  7.2. ICU mortality associated with VAP  7.3. In-hospital mortality associated with VAP | Meta-analysis | Analysis 7.1. Comparison 7. Cases (VAP) versus controls (non-VAP), Outcome 2. VAP as predictor of patient mortality (Higher in non-VAP or higher in VAP?)  Analysis 7.2. Comparison 7. Cases (VAP) versus controls (non-VAP), Outcome 3. ICU mortality associated with VAP (Higher in non-VAP or higher in VAP?)  Analysis 7.3. Comparison 7. Cases (VAP) versus controls (non-VAP), Outcome 4. In-hospital mortality associated with VAP (Higher in non-VAP or higher in VAP?) |

| **Table S7** Continued | | |
| --- | --- | --- |
| **Research question** | **Analysis type** | **Planned data analysis** |
| 1. Do patients with VAP have longer duration of MV compared to patients without VAP? | Meta-analysis | Analysis 8. Comparison 7. Cases (VAP) versus controls (non-VAP), Outcome 5. Duration of MV, in days (Longer in non-VAP or longer in VAP?) |
| 1. Do patients with VAP have increased in ICU LOS compared to patients without VAP? | Meta-analysis | Analysis 9. Comparison 7. Cases (VAP) versus controls (non-VAP), Outcome 6. ICU LOS, in days (Longer in non-VAP or longer in VAP?) |
| 1. Do patients with VAP have increased in hospital LOS compared to patients without VAP? | Meta-analysis | Analysis 10. Comparison 7. Cases (VAP) versus controls (non-VAP), Outcome 7. LOS in hospital, in days (Longer in non-VAP or longer in VAP?) |
| 1. Do patients with VAP have higher microbial colonization compared to patients without VAP? | Meta-analysis | Analysis 11. Comparison 7. Cases (VAP) versus controls (non-VAP), Outcome 8. Microbial colonization (Higher in non-VAP or higher in VAP?) |
| 1. Do patients with VAP have higher antibiotic cost compared to patients without VAP? | Meta-analysis | Analysis 12. Comparison 7. Cases (VAP) versus controls (non-VAP), Outcome 9. Cost of antibiotic treatment of VAP (Higher in non-VAP or higher in VAP?) |
| 1. Do patients with VAP have higher hospital cost compared to patients without VAP? | Meta-analysis | Analysis 13. Comparison 7. Cases (VAP) versus controls (non-VAP), Outcome 10. Hospitalization cost (Higher in non-VAP or higher in VAP?) |
| *Note:* ICU, intensive care unit; IQR, interquartile range; LOS, length of stay; MV, mechanical ventilation; RRI, relative risk increase; SD, standard deviation; VAP, ventilator-associated pneumonia. | | |
