# Supplementary material for: Clinical epidemiology and outcomes of ventilator-associated pneumonia in critically ill adult patients: protocol for a large-scale systematic review and planned meta-analysis
Source: Syst Rev. 2019 Jul 20;8:180. doi: 10.1186/s13643-019-1080-y (PMC6642735; doi:10.1186/s13643-019-1080-y)
Supplement: Supplementary file 3 — Flow diagrams. (DOCX 402 kb) [file 13643_2019_1080_MOESM3_ESM.docx]

**Fig. 1.** The Search Strategy Decision Algorithm

*Notes*:

* If the study authors used non-specific terms, such as “pneumonia”, “nosocomial pneumonia”, “nosocomial infection”, “device-associated infection (DAI)”, “healthcare-associated infection (HAI or HCAI)”, “device-associated hospital-acquired infection (DAI-HAI)”, and “hospital-acquired pneumonia (HAP)”, determine whether the study populations were intubated/ventilated. Exclude ventilator-associated tracheobronchitis (VAT), community-acquired pneumonia (CAP) or infections with non-pulmonary focus (e.g., catheter-associated UTI, surgical site infection, central line associated bloodstream infection, meningitis, peritonitis, bacteremia, and the like; UNLESS co-infection exists (e.g., VAP with non-VAP-related infections).

** Please refer to PhD Screening Checklist.docx

*** Reasons for exclusion include clinical practice guidelines/recommendations, summary report, reviews, protocols, quasi-experiments, ecological study, cross-sectional study, case series/case reports, diagnostics, descriptive surveys, tool development study, qualitative study/mixed methods, preliminary report, research methods, editorial, commentaries/letters/views/opinion, abstract, symposium/conference paper, registered trials, book chapters, recurrent VAP, not ICU, not adult, hypothetical cohort, animal models, not English, no eligible control, not microbiologically confirmed VAP, confirmed VAP not specified (use of *Clinical Pulmonary Infection Score*), not meeting MV cutoff point (*<48h*), not meeting age cutoff (<15y), ventilated patients not specified, brief report, articles not eligible, no VAP data, VAP episodes cannot be clarified, full-text not available, and duplicate full-text.

When marking citations, use number one (1) in Excel spreadsheet file.

**Fig. 2.** PRISMA flow diagram of study selection for inclusion in meta-analysis

**Fig. 3.** Meta-analysis decision-making algorithm (VAP epidemiological indexes)

*Notes:* CI, confidence intervals; ICU, intensive care unit; LOS, length of stay; MD, mean difference; MV, mechanical ventilation; OR, odds ratio; VAP, ventilator-associated pneumonia
