# Supplementary material for: Clinical epidemiology and outcomes of ventilator-associated pneumonia in critically ill adult patients: protocol for a large-scale systematic review and planned meta-analysis
Source: Syst Rev. 2019 Jul 20;8:180. doi: 10.1186/s13643-019-1080-y (PMC6642735; doi:10.1186/s13643-019-1080-y)
Supplement: Supplementary file 4 — Data forms. (DOCX 161 kb) [file 13643_2019_1080_MOESM4_ESM.docx]

| **Appendix 1.** Screening Checklist | | | | | |
| --- | --- | --- | --- | --- | --- |
| General information | | | | | |
| Date started: |  | | | | |
| Date completed: |  | | | | |
| Name/ID of person extracting the data: |  | | | | |
| Data extractor contact details: |  | | | | |
| Report title:  *(Title of paper/abstract/report that data are extracted from)* |  | | | | |
| Report PMID *(PubMed ID)* |  | | | | |
| Last name of first author: |  | | | | |
| Journal *(NLM title abbreviation):* |  | | | | |
| Year published: |  | | | | |
| Country of origin: |  | | | | |
| Report author contact details: |  | | | | |
| Publication type  *(e.g., Full text, full report, abstract, etc.)* |  | | | | |
| Assigned study ID: |  | | | | |
| If conflict of interest exists, please do NOT proceed. Check the ‘Conflict’ box under ‘Review Decision’. | | | | | |
| Review eligibility criteria (please refer to *Table 1*) | |  |  |  |  |
| Study characteristics | Inclusion criteria *(Insert eligibility criteria for each characteristic as defined in the review protocol)* | Yes | No | Unsure | Location in text  *(Page, figure, table)* |
| Types of study designs:  *(Choose one only)* | Prospective cohort study |  |  |  |  |
|  | Was the study design: |  |  |  |  |
|  | Randomized controlled trial (RCT); |  |  |  |  |
|  | Prospective cohort study or prospective analysis; |  |  |  |  |
|  | Retrospective cohort study or retrospective analysis; |  |  |  |  |
|  | Nested case-control study; |  |  |  |  |
|  | Case-control study: similar or equivalent controls (patient status, timing, etc.)? |  |  |  |  |
|  | Case-control study: unclear or different |  |  |  |  |
| Types of participants: | Did the study authors include about adult patients (15 years or older) requiring intubation/mechanical ventilation ≥48h? |  |  |  |  |
|  | Did the study authors include case patients with corresponding comparison groups (controls) as per protocol definition? |  |  |  |  |
|  | Did the study authors report patients with or without pre-existing lung infections at ICU admission or before intubation or initiation of mechanical ventilation?^[[1]](#footnote-1)^ |  |  |  |  |
| Types of exposures:  *(Please see ‘Table for types of VAP exposures’ and select all that apply)* | Did the study authors report any of the following factors: |  |  |  |  |
|  | Host- or patient-related factors; |  |  |  |  |
|  | Intervention- or treatment-related factors; |  |  |  |  |
|  | Device-related factors; |  |  |  |  |
|  | Personnel-related factors; |  |  |  |  |
|  | Environmental-related factors; |  |  |  |  |
|  | Others? |  |  |  |  |
| Types of outcome measures: | Primary outcome: Did the study authors only include patients with initial episode of microbiologically confirmed VAP? |  |  |  |  |
| Types of outcome measures:  *(Please select all that apply)* | Did the study authors report any of the following secondary outcome measures: |  |  |  |  |
|  | Duration of mechanical ventilation; |  |  |  |  |
|  | Length of ICU or hospital stay; |  |  |  |  |
|  | Mortality among VAP cases and controls; |  |  |  |  |
|  | Microbial profiles; |  |  |  |  |
|  | Antibiotic cost; |  |  |  |  |
|  | Hospitalization cost? |  |  |  |  |
| Types of setting | Was the study conducted only in ICU? |  |  |  |  |
| Review Decision:   - Include - Include relevant results only - Exclude - Awaiting assignment | Reasons for exclusion *(Please refer to eligibility criteria. Examples: Not microbiologically confirmed; not English; no eligible control; full-text, not available)*: | | | | |
| Do NOT proceed to Data Extraction if you answer ‘NO’ to any of the above inclusion criteria (Except for sections under *Types of exposures* and *secondary outcome measures*). ‘UNSURE’ answers need to be validated. If study is to be excluded, record the information to be inserted into ‘Table of excluded studies.’  DO NOT PROCEED TO DATA EXTRACTION IF STUDY EXCLUDED FROM REVIEW | | | | | |

**INSTRUCTIONS: Read the included article and carefully extract the needed data for review. Please refer to the study protocol.**

| **Appendix 2.** Data Extraction Form | | | | |
| --- | --- | --- | --- | --- |
| **Study objectives** |  | | | **Location in text**  *(Page number, section, paragraph, figure, table)* |
| **Study objective no. 1:** |  | | |  |
| **Study objective no. 2:** |  | | |  |
| **Study objective no. 3:** |  | | |  |
| **Study objective no. 4:** |  | | |  |
| **Study objective no. 5:** |  | | |  |
|  | | | | |
| **Population & setting** |  | | | **Location in text**  *(Page number, section, paragraph, figure, table)* |
| **Type of patients/setting:**  *(e.g. Medical ICU patients, surgical ICU patients, med-surg ICU patients, neuro ICU patients, neuro-surgical ICU, cardiac ICU patients, cardiac surgical patients, trauma ICU, surgical trauma ICU, burn ICU, and combined or mixed-type)* |  | | |  |
| **Number of participants enrolled in the study:** |  | | |  |
| **Demographic & clinical characteristics of study cases and controls** *(Please list down the mentioned demographic and clinical characteristics: e.g., mean age for VAP and non-VAP patients)* |  | | |  |
|  | | | | |
| **Enrollment period or length of follow-up** |  | | | **Location in text**  *(Page number, section, paragraph, figure, table)* |
| **Dates:**  *(e.g. mm/dd/yyy)* |  | | |  |
| **Number of dropouts** |  | | |  |
| **Non-response rate** |  | | |  |
|  | | | | |
| **Exposure definition** |  | | |  |
| **Sources of information on exposure**  *(Please identify the source of exposure)* |  | | |  |
|  |  | | |  |
| **Sources of cases and control** |  | | |  |
| *(Please identify the source of cases and controls: e.g. clinical or medical records, database, VAP report, etc.)* |  | | |  |
|  | | | | |
| **Outcome definition** | | | | **Location in text**  *(Page number, section, paragraph, figure, table)* |
| **VAP diagnostic criteria (as stated by study authors):**  *(e.g. clinical criteria, radiographic and microbial examinations using BAL fluid, PSB, etc.)* |  | | |  |
| **Ratio of VAP and non-VAP patients** |  | | |  |
| **Number of VAP in patients ventilated for ≤96 hours** |  | | |  |
| **Number of VAP in patients ventilated for >96 hours** |  | | |  |
| **VAP episodes per 1000 ventilation days** |  | | |  |
| **Ventilator days** |  | | |  |
| **VAP onset** |  | | |  |
| **Duration of mechanical ventilation in VAP and non-VAP patients** |  | | |  |
| **Length of ICU stay in VAP and non-VAP patients** |  | | |  |
| **Length of hospital stay in VAP and non-VAP patients** |  | | |  |
|  | | | | |
| **Methodology** |  |  |  | **Location in text**  *(Page number, section, paragraph, figure, table)* |
| **Matching criteria used**  *(e.g. Age)* |  | | |  |
| **Identify adjustments for confounding variables.**  *(e.g., Age)* |  | | |  |
|  |  |  |  |  |
| **Risk factors with risk estimates** |  |  |  | **Location in text**  *(Page number, section, paragraph, figure, table)* |
| **Risk estimates**  *(Please list down ALL reported risk assessments and include number of exposed and non-exposed patients or number of exposed patients and total population per risk factor with corresponding risk estimates. Example of risk estimates: RR, OR, HR with 95% CI)*  **Example:**  Risk factor: **Re-intubation**  Exposed cases: 12  Total cases: 105  Exposed controls: 40  Total controls: 1583  Risk Estimates (95% CI): 4.98 (2.53, 9.81) | **Risk factors for VAP:**  **Risk factors for mortality:**  **Risk factors for increased duration of MV:**  **Risk factors for increased ICU LOS:**  **Risk factors for hospital LOS:**  **Risk factors for specific microorganisms:** | | |  |
|  | | | | |
| **Mortality assessment** | | | | **Location in text**  *(Page number, section, paragraph, figure, table)* |
| **Number of mortality (all-causes)**  *(Please list down how many number of patients died during the study period)* | **VAP:**  **Non-VAP:** | | |  |
| **Number of mortality related to VAP**  *(Please list down how many number of VAP cases died during the study period)* | **ICU mortality:**  **Hospital mortality:**  **28-day mortality**  **1-year mortality:** | | |  |
| **VAP as attributable mortality**  *(Please identify if the study attributed VAP to patient mortality)* |  | | |  |
|  | | | | |
| **Costs and Isolated pathogens** | | | | **Location in text**  *(Page number, section, paragraph, figure, table)* |
|  |  | | |  |
| **Healthcare costs (in US dollars) incurred by VAP and non-VAP patients** |  | | |  |
| **Antibiotic costs (in US dollars) incurred by VAP and non-VAP patients** |  | | |  |
| **Isolated pathogens among survivors** *(Please list down the number and percentage of isolated pathogens)* |  | | |  |
| **Isolated pathogens among non-survivors** *(Please list down the number and percentage of isolated pathogens)* |  | | |  |
|  | | | | |
| **Quality assessment** | | | |  |
|  |  | | |  |
| *(Please indicate the quality assessment using either Risk of Bias for RCTs or NOS score for observational studies)* |  | | |  |
|  | | | |  |
| **References details** | | | | **Location in text**  *(Page number, section, paragraph, figure, table)* |
|  |  | | |  |
| *(Please highlight (in red text) relevant citations or similar articles for possible inclusion)* |  | | |  |
| **----End----** | | | | |

Assessed by: Validated by:

**John Mark M. Gutiérrez Name**

*Principal Investigator Data Extractor*

Noted by:

**Dr. Annabelle R. Borromeo**

*PhD Adviser*

*__/__/____*

| **Appendix 3**. The Quality in Prognosis Studies (QUIPS) Tool | | | | |
| --- | --- | --- | --- | --- |
| **First author (publication year)** |  | | | |
| **Study title** |  | | | |
| **Study reviewer** |  | | | |
|  | | | | |
| **Biases** | **Issues to consider for judging overall rating of “Risk of bias”** | **Study methods & comments** | **Rating of reporting** | **Rating of “Risk of bias”** |
| Instructions to assess the risk of each potential bias: | These issues will guide your thinking and judgment about the overall risk of bias within each of the 6 domains. Some 'issues' may not be relevant to the specific study or the review research question. These issues are taken together to inform the overall judgment of potential bias for each of the 6 domains. | Provide comments or text excerpts in the white boxes below, as necessary, to facilitate the consensus process that will follow. | Click on each of the blue cells and choose from the drop down menu to rate the adequacy of reporting as yes, partial, no or unsure. | Click on the green cells; choose from the drop-down menu to rate potential risk of bias for each of the 6 domains as High, Moderate, or Low considering all relevant issues. |
| **1. Study Participation** | **Goal: To judge the risk of selection bias (likelihood that relationship between *PF* and *outcome* is different for participants and eligible non-participants).** |  |  |  |
| *Source of target population* | The source population or population of interest is adequately described for key characteristics (LIST). |  |  |  |
| *Method used to identify population* | The sampling frame and recruitment are adequately described, including methods to identify the sample sufficient to limit potential bias (number and type used, e.g., referral patterns in health care). |  |  |  |
| *Recruitment period* | Period of recruitment is adequately described. |  |  |  |
| *Place of recruitment* | Place of recruitment (setting and geographic location) are adequately described. |  |  |  |
| *Inclusion and exclusion criteria* | Inclusion and exclusion criteria are adequately described (e.g., including explicit diagnostic criteria or |  |  |  |
| *Adequate study participation* | There is adequate participation in the study by eligible individuals |  |  |  |
| *Baseline characteristics* | The baseline study sample (i.e., individuals entering the study) is adequately described for key characteristics (LIST). |  |  |  |
| **Summary Study participation** | **The study sample represents the population of interest on key characteristics, sufficient to limit potential bias of the observed relationship between PF and outcome.** |  |  |  |
|  | | | | |
| **2. Study Attrition** | **Goal: To judge the risk of attrition bias (likelihood that relationship between *PF* and *outcome* are different for completing and non-completing participants).** |  |  |  |
| *Proportion of baseline sample available for analysis* | Response rate (i.e., proportion of study sample completing the study and providing outcome data) is adequate. |  |  |  |
| *Attempts to collect information on participants who dropped out* | Attempts to collect information on participants who dropped out of the study are described. |  |  |  |
| *Reasons and potential impact of subjects lost to follow-up* | Reasons for loss to follow-up are provided. |  |  |  |
| *Outcome and prognostic factor information on those lost to follow-up* | Participants lost to follow-up are adequately described for key characteristics (LIST). |  |  |  |
|  | There are no important differences between key characteristics (LIST) and outcomes in participants who completed the study and those who did not. |  |  |  |
| **Study Attrition Summary** | **Loss to follow-up (from baseline sample to study population analyzed) is not associated with key characteristics (i.e., the study data adequately represent the sample) sufficient to limit potential bias to the observed relationship between PF and outcome.** |  |  |  |
|  | | | | |
| **3. Prognostic Factor Measurement** | **Goal: To judge the risk of measurement bias related to how PF was measured (differential measurement of PF related to the level of outcome).** |  |  |  |
| *Definition of the PF* | A clear definition or description of 'PF' is provided (e.g., including dose, level, duration of exposure, and clear specification of the method of measurement). |  |  |  |
| *Valid and Reliable Measurement of PF* | Method of PF measurement is adequately valid and reliable to limit misclassification bias (e.g., may include relevant outside sources of information on measurement properties, also characteristics, such as blind measurement and limited reliance on recall). |  |  |  |
|  | Continuous variables are reported or appropriate cut-points (i.e., not data-dependent) are used. |  |  |  |
| *Method and Setting of PF Measurement* | The method and setting of measurement of PF is the same for all study participants. |  |  |  |
| *Proportion of data on PF available for analysis* | Adequate proportion of the study sample has complete data for PF variable. |  |  |  |
| *Method used for missing data* | Appropriate methods of imputation are used for missing 'PF' data. |  |  |  |
| **PF Measurement Summary** | ***PF* is adequately measured in study participants to sufficiently limit potential bias.** |  |  |  |
|  | | | | |
| **Appendix 3**. Continuation. | | | | |
| **Biases** | **Issues to consider for judging overall rating of “Risk of bias”** | **Study methods & comments** | **Rating of reporting** | **Rating of “Risk of bias”** |
| Instructions to assess the risk of each potential bias: | These issues will guide your thinking and judgment about the overall risk of bias within each of the 6 domains. Some 'issues' may not be relevant to the specific study or the review research question. These issues are taken together to inform the overall judgment of potential bias for each of the 6 domains. | Provide comments or text excerpts in the white boxes below, as necessary, to facilitate the consensus process that will follow. | Click on each of the blue cells and choose from the drop down menu to rate the adequacy of reporting as yes, partial, no or unsure. | Click on the green cells; choose from the drop-down menu to rate potential risk of bias for each of the 6 domains as High, Moderate, or Low considering all relevant issues. |
| **4. Outcome Measurement** | **Goal: To judge the risk of bias related to the measurement of outcome (differential measurement of outcome related to the baseline level of PF).** |  |  |  |
| *Definition of the Outcome* | A clear definition of outcome is provided, including duration of follow-up and level and extent of the outcome construct. |  |  |  |
| *Valid and Reliable Measurement of Outcome* | The method of outcome measurement used is adequately valid and reliable to limit misclassification bias (e.g., may include relevant outside sources of information on measurement properties, also characteristics, such as blind measurement and confirmation of outcome with valid and reliable test). |  |  |  |
| *Method and Setting of Outcome Measurement* | The method and setting of outcome measurement is the same for all study participants. |  |  |  |
| **Outcome Measurement Summary** | ***Outcome of interest* is adequately measured in study participants to sufficiently limit potential bias.** |  |  |  |
|  | | | | |
| **5. Study Confounding** | **Goal: To judge the risk of bias due to confounding (i.e. the effect of PF is distorted by another factor that is related to PF and outcome).** |  |  |  |
| *Important Confounders Measured* | All important confounders, including treatments (key variables in conceptual model: LIST), are measured. |  |  |  |
| *Definition of the confounding factor* | Clear definitions of the important confounders measured are provided (e.g., including dose, level, and duration of exposures). |  |  |  |
| *Valid and Reliable Measurement of Confounders* | Measurement of all important confounders is adequately valid and reliable (e.g., may include relevant outside sources of information on measurement properties, also characteristics, such as blind measurement and limited reliance on recall). |  |  |  |
| *Method and Setting of Confounding Measurement* | The method and setting of confounding measurement are the same for all study participants. |  |  |  |
| *Method used for missing data* | Appropriate methods are used if imputation is used for missing confounder data. |  |  |  |
| *Appropriate Accounting for Confounding* | Important potential confounders are accounted for in the study design (e.g., matching for key variables, stratification, or initial assembly of comparable groups). |  |  |  |
|  | Important potential confounders are accounted for in the analysis (i.e., appropriate adjustment). |  |  |  |
| **Study Confounding Summary** | **Important potential confounders are appropriately accounted for, limiting potential bias with respect to the relationship between *PF* and *outcome*.** |  |  |  |
|  | | | | |
| **6. Statistical Analysis and Reporting** | **Goal: To judge the risk of bias related to the statistical analysis and presentation of results.** |  |  |  |
| *Presentation of analytical strategy* | There is sufficient presentation of data to assess the adequacy of the analysis. |  |  |  |
| *Model development strategy* | The strategy for model building (i.e., inclusion of variables in the statistical model) is appropriate and is based on a conceptual framework or model. |  |  |  |
|  | The selected statistical model is adequate for the design of the study. |  |  |  |
| *Reporting of results* | There is no selective reporting of results. |  |  |  |
| **Statistical Analysis and Presentation Summary** | **The statistical analysis is appropriate for the design of the study, limiting potential for presentation of invalid or spurious results.** |  |  |  |
|  | | | | |
| Abbreviation: PF, prognostic factor.  Adapted from Hayden and colleagues, with permission. | | | | |

**Date Completed:**

1. This is not part of the review inclusion criteria. The data obtained herein will be used in sensitivity analysis. [↑](#footnote-ref-1)
